# Supplementary material for: Modeling glioblastoma heterogeneity as a dynamic network of cell states
Source: Mol Syst Biol. 2021 Sep 16;17(9):e10105. doi: 10.15252/msb.202010105 (PMC8444284; doi:10.15252/msb.202010105)
Supplement: Supplementary file 6 — Source Data for Figure 5 [file MSB-17-e10105-s004.zip › Figure5A_sourcedata/GSEA_3017/nosigdb_stateC.GseaPreranked.1622795002785/RICHARDS_NOWAKOWSKI_SCIENCE_2017_TRG_UPREG.html]

Details for gene set RICHARDS\_NOWAKOWSKI\_SCIENCE\_2017\_TRG\_UPREG[GSEA]

|  || Dataset | state13017 |
| Phenotype | NoPhenotypeAvailable |
| Upregulated in class | na\_neg |
| GeneSet | RICHARDS\_NOWAKOWSKI\_SCIENCE\_2017\_TRG\_UPREG |
| Enrichment Score (ES) | -0.3564925 |
| Normalized Enrichment Score (NES) | -2.6370623 |
| Nominal p-value | 0.0 |
| FDR q-value | 0.0 |
| FWER p-Value | 0.0 |
Table: GSEA Results Summary

  

Fig 1: Enrichment plot: RICHARDS\_NOWAKOWSKI\_SCIENCE\_2017\_TRG\_UPREG      
 Profile of the Running ES Score & Positions of GeneSet Members on the Rank Ordered List

  

| PROBE | GENE SYMBOL | GENE\_TITLE | RANK IN GENE LIST | RANK METRIC SCORE | RUNNING ES | CORE ENRICHMENT || 1 | MAGI1 |  |  | 6 | 0.652 | -0.0006 | No |
| 2 | ATAD2 |  |  | 7 | 0.649 | 0.0118 | No |
| 3 | FAM111A |  |  | 13 | 0.576 | 0.0119 | No |
| 4 | ZBTB20 |  |  | 30 | 0.482 | -0.0135 | No |
| 5 | CDCA7L |  |  | 37 | 0.453 | -0.0178 | No |
| 6 | GMNN |  |  | 38 | 0.453 | -0.0092 | No |
| 7 | ETV1 |  |  | 44 | 0.438 | -0.0117 | No |
| 8 | PHIP |  |  | 61 | 0.387 | -0.0389 | No |
| 9 | POLD3 |  |  | 76 | 0.367 | -0.0621 | No |
| 10 | GINS2 |  |  | 83 | 0.360 | -0.0682 | No |
| 11 | DUT |  |  | 87 | 0.356 | -0.0679 | No |
| 12 | CKB |  |  | 102 | 0.335 | -0.0918 | No |
| 13 | MCM3 |  |  | 114 | 0.324 | -0.1094 | No |
| 14 | HAT1 |  |  | 121 | 0.318 | -0.1163 | No |
| 15 | STAG2 |  |  | 128 | 0.309 | -0.1234 | No |
| 16 | ASCL1 |  |  | 145 | 0.290 | -0.1525 | No |
| 17 | CCND2 |  |  | 149 | 0.288 | -0.1535 | No |
| 18 | HELLS |  |  | 171 | 0.271 | -0.1937 | No |
| 19 | C19orf48 |  |  | 173 | 0.271 | -0.1907 | No |
| 20 | NKAIN3 |  |  | 175 | 0.268 | -0.1877 | No |
| 21 | SNCAIP |  |  | 182 | 0.264 | -0.1957 | No |
| 22 | FANCL |  |  | 185 | 0.261 | -0.1950 | No |
| 23 | MCM4 |  |  | 189 | 0.259 | -0.1966 | No |
| 24 | SNTG1 |  |  | 193 | 0.252 | -0.1983 | No |
| 25 | CAST |  |  | 205 | -0.253 | -0.2172 | No |
| 26 | SHC3 |  |  | 206 | -0.253 | -0.2124 | No |
| 27 | ARHGAP12 |  |  | 207 | -0.254 | -0.2076 | No |
| 28 | PGAM1 |  |  | 218 | -0.258 | -0.2243 | No |
| 29 | FERMT2 |  |  | 227 | -0.259 | -0.2366 | No |
| 30 | IGF2BP2 |  |  | 231 | -0.260 | -0.2382 | No |
| 31 | MBNL2 |  |  | 233 | -0.260 | -0.2354 | No |
| 32 | ZYX |  |  | 236 | -0.261 | -0.2347 | No |
| 33 | SAT1 |  |  | 241 | -0.262 | -0.2384 | No |
| 34 | LIMS1 |  |  | 244 | -0.262 | -0.2377 | No |
| 35 | PCGF5 |  |  | 252 | -0.264 | -0.2478 | No |
| 36 | NPAS3 |  |  | 256 | -0.265 | -0.2493 | No |
| 37 | ID4 |  |  | 261 | -0.267 | -0.2528 | No |
| 38 | GATM |  |  | 272 | -0.270 | -0.2693 | No |
| 39 | HSD17B12 |  |  | 277 | -0.271 | -0.2728 | No |
| 40 | HEATR5A |  |  | 278 | -0.271 | -0.2676 | No |
| 41 | DTNA |  |  | 281 | -0.271 | -0.2668 | No |
| 42 | TGFB2 |  |  | 285 | -0.272 | -0.2681 | No |
| 43 | SQLE |  |  | 293 | -0.274 | -0.2780 | No |
| 44 | PHLDA1 |  |  | 295 | -0.274 | -0.2750 | No |
| 45 | SPAG16 |  |  | 300 | -0.277 | -0.2783 | No |
| 46 | HERPUD1 |  |  | 302 | -0.277 | -0.2752 | No |
| 47 | CNKSR3 |  |  | 305 | -0.280 | -0.2742 | No |
| 48 | NAMPT |  |  | 306 | -0.280 | -0.2689 | No |
| 49 | SLC1A3 |  |  | 307 | -0.281 | -0.2636 | No |
| 50 | CREB5 |  |  | 309 | -0.281 | -0.2604 | No |
| 51 | LIFR |  |  | 311 | -0.282 | -0.2572 | No |
| 52 | NUDT4 |  |  | 321 | -0.285 | -0.2712 | No |
| 53 | MVD |  |  | 333 | -0.288 | -0.2895 | No |
| 54 | PPP1R15A |  |  | 342 | -0.293 | -0.3012 | No |
| 55 | LRP1 |  |  | 366 | -0.304 | -0.3451 | No |
| 56 | PDPN |  |  | 372 | -0.307 | -0.3500 | No |
| 57 | LMNA |  |  | 376 | -0.308 | -0.3506 | Yes |
| 58 | FOS |  |  | 377 | -0.309 | -0.3448 | Yes |
| 59 | SLC2A1 |  |  | 383 | -0.311 | -0.3496 | Yes |
| 60 | IGFBP5 |  |  | 384 | -0.311 | -0.3437 | Yes |
| 61 | SAMD4A |  |  | 388 | -0.313 | -0.3443 | Yes |
| 62 | RHOB |  |  | 391 | -0.316 | -0.3426 | Yes |
| 63 | CETN2 |  |  | 392 | -0.317 | -0.3365 | Yes |
| 64 | PLTP |  |  | 397 | -0.319 | -0.3391 | Yes |
| 65 | ZFP36L2 |  |  | 401 | -0.321 | -0.3395 | Yes |
| 66 | FGFR3 |  |  | 403 | -0.322 | -0.3355 | Yes |
| 67 | GADD45A |  |  | 405 | -0.323 | -0.3316 | Yes |
| 68 | TPM1 |  |  | 407 | -0.327 | -0.3275 | Yes |
| 69 | SIPA1L1 |  |  | 412 | -0.329 | -0.3299 | Yes |
| 70 | DDX21 |  |  | 413 | -0.331 | -0.3236 | Yes |
| 71 | HMGCR |  |  | 418 | -0.334 | -0.3259 | Yes |
| 72 | LDLR |  |  | 419 | -0.334 | -0.3195 | Yes |
| 73 | CALD1 |  |  | 420 | -0.335 | -0.3132 | Yes |
| 74 | SLC2A3 |  |  | 422 | -0.336 | -0.3089 | Yes |
| 75 | RHOQ |  |  | 426 | -0.338 | -0.3090 | Yes |
| 76 | KLF6 |  |  | 429 | -0.339 | -0.3069 | Yes |
| 77 | SNHG16 |  |  | 431 | -0.340 | -0.3026 | Yes |
| 78 | REXO2 |  |  | 432 | -0.342 | -0.2961 | Yes |
| 79 | INSIG1 |  |  | 434 | -0.342 | -0.2917 | Yes |
| 80 | VEGFA |  |  | 436 | -0.345 | -0.2873 | Yes |
| 81 | WWTR1 |  |  | 437 | -0.345 | -0.2808 | Yes |
| 82 | FAT1 |  |  | 441 | -0.346 | -0.2807 | Yes |
| 83 | RAB31 |  |  | 445 | -0.349 | -0.2805 | Yes |
| 84 | HSPA5 |  |  | 447 | -0.350 | -0.2760 | Yes |
| 85 | MYL12A |  |  | 448 | -0.350 | -0.2694 | Yes |
| 86 | BCAN |  |  | 449 | -0.351 | -0.2627 | Yes |
| 87 | IDI1 |  |  | 451 | -0.353 | -0.2581 | Yes |
| 88 | NEK6 |  |  | 453 | -0.355 | -0.2535 | Yes |
| 89 | DKK3 |  |  | 454 | -0.356 | -0.2468 | Yes |
| 90 | RHOC |  |  | 455 | -0.359 | -0.2399 | Yes |
| 91 | SLITRK2 |  |  | 458 | -0.361 | -0.2374 | Yes |
| 92 | FLRT3 |  |  | 461 | -0.363 | -0.2348 | Yes |
| 93 | SERPINB6 |  |  | 463 | -0.367 | -0.2300 | Yes |
| 94 | B2M |  |  | 468 | -0.371 | -0.2316 | Yes |
| 95 | HES1 |  |  | 470 | -0.373 | -0.2266 | Yes |
| 96 | CYP51A1 |  |  | 476 | -0.378 | -0.2302 | Yes |
| 97 | HBEGF |  |  | 480 | -0.386 | -0.2294 | Yes |
| 98 | VCL |  |  | 484 | -0.388 | -0.2285 | Yes |
| 99 | C1orf61 |  |  | 491 | -0.396 | -0.2339 | Yes |
| 100 | FABP7 |  |  | 495 | -0.411 | -0.2326 | Yes |
| 101 | LDHA |  |  | 498 | -0.412 | -0.2291 | Yes |
| 102 | TGIF1 |  |  | 502 | -0.421 | -0.2275 | Yes |
| 103 | JUN |  |  | 503 | -0.425 | -0.2194 | Yes |
| 104 | SCD |  |  | 504 | -0.431 | -0.2113 | Yes |
| 105 | F3 |  |  | 505 | -0.432 | -0.2030 | Yes |
| 106 | DBI |  |  | 514 | -0.440 | -0.2119 | Yes |
| 107 | IRS2 |  |  | 516 | -0.444 | -0.2057 | Yes |
| 108 | PTN |  |  | 517 | -0.446 | -0.1972 | Yes |
| 109 | MSMO1 |  |  | 525 | -0.460 | -0.2036 | Yes |
| 110 | HMGCS1 |  |  | 529 | -0.466 | -0.2012 | Yes |
| 111 | IFITM3 |  |  | 533 | -0.477 | -0.1986 | Yes |
| 112 | ITGA6 |  |  | 535 | -0.479 | -0.1916 | Yes |
| 113 | CRYAB |  |  | 536 | -0.485 | -0.1824 | Yes |
| 114 | TTYH1 |  |  | 540 | -0.496 | -0.1795 | Yes |
| 115 | HOPX |  |  | 546 | -0.513 | -0.1805 | Yes |
| 116 | DANCR |  |  | 547 | -0.516 | -0.1707 | Yes |
| 117 | SNHG3 |  |  | 549 | -0.518 | -0.1630 | Yes |
| 118 | PLAT |  |  | 556 | -0.538 | -0.1657 | Yes |
| 119 | CREM |  |  | 562 | -0.563 | -0.1658 | Yes |
| 120 | ANXA2 |  |  | 563 | -0.567 | -0.1550 | Yes |
| 121 | TNC |  |  | 564 | -0.572 | -0.1442 | Yes |
| 122 | MT2A |  |  | 566 | -0.575 | -0.1354 | Yes |
| 123 | SPARC |  |  | 567 | -0.575 | -0.1245 | Yes |
| 124 | FGFBP3 |  |  | 573 | -0.644 | -0.1230 | Yes |
| 125 | GFAP |  |  | 574 | -0.659 | -0.1105 | Yes |
| 126 | VIM |  |  | 578 | -0.674 | -0.1041 | Yes |
| 127 | NEAT1 |  |  | 579 | -0.683 | -0.0912 | Yes |
| 128 | CLU |  |  | 581 | -0.690 | -0.0802 | Yes |
| 129 | CYR61 |  |  | 584 | -0.714 | -0.0709 | Yes |
| 130 | ATP1B2 |  |  | 585 | -0.714 | -0.0574 | Yes |
| 131 | CA12 |  |  | 586 | -0.717 | -0.0437 | Yes |
| 132 | CTGF |  |  | 588 | -0.782 | -0.0310 | Yes |
| 133 | PMP22 |  |  | 590 | -0.837 | -0.0173 | Yes |
| 134 | FABP5 |  |  | 596 | -1.477 | -0.0000 | Yes |
Table: GSEA details [plain text format]

  

Fig 2: RICHARDS\_NOWAKOWSKI\_SCIENCE\_2017\_TRG\_UPREG: Random ES distribution      
 Gene set null distribution of ES for **RICHARDS\_NOWAKOWSKI\_SCIENCE\_2017\_TRG\_UPREG**
